# Induction of subject-ventilator asynchrony by variation of respiratory parameters in a lung injury model in pigs

**DOI:** 10.1186/s12931-024-02984-y

**Published:** 2024-10-03

**Authors:** Xi Ran, Martin Scharffenberg, Jakob Wittenstein, Mark Leidermann, Andreas Güldner, Thea Koch, Marcelo Gama de Abreu, Robert Huhle

**Affiliations:** 1 Medical Research Center, Chongqing General Hospital, Chongqing University, Chongqing, China; 2 Department of Anesthesiology and Intensive Care Medicine, Pulmonary Engineering Group, Faculty of Medicine and University Hospital Carl Gustav Carus, TUD Dresden University of Technology, Dresden, Germany; 3 Department of Intensive Care and Resuscitation, Anesthesiology Institute, Cleveland Clinic, Cleveland, OH USA; 4 Department of Outcomes Research, Anesthesiology Institute, Cleveland Clinic, Cleveland, OH USA

**Keywords:** Subject-ventilator asynchrony, Ineffective efforts, Auto-triggering, Double-triggering, Large animal model

## Abstract

**Background:**

Subject-ventilator asynchrony (SVA) was shown to be associated with negative clinical outcomes. To elucidate pathophysiology pathways and effects of SVA on lung tissue histology a reproducible animal model of artificially induced asynchrony was developed and evaluated.

**Methods:**

Alterations in ventilator parameters were used to induce the three main types of asynchrony: ineffective efforts (IE), auto-triggering (AT), and double-triggering (DT). Airway flow and pressure, as well as oesophageal pressure waveforms, were recorded, asynchrony cycles were manually classified and the asynchrony index (AIX) was calculated. Bench tests were conducted on an active lung simulator with ventilator settings altered cycle by cycle. The developed algorithm was evaluated in three pilot experiments and a study in pigs ventilated for twelve hours with AIX = 25%.

**Results:**

IE and AT were induced reliably and fail-safe by end-expiratory hold and adjustment of respiratory rate, respectively. DT was provoked using airway pressure ramp prolongation, however not controlled specifically in the pilots. In the subsequent study, an AIX = 28.8% [24.0%-34.4%] was induced and maintained over twelve hours.

**Conclusions:**

The method allows to reproducibly induce and maintain three clinically relevant types of SVA observed in ventilated patients and may thus serve as a useful tool for future investigations on cellular and inflammatory effects of asynchrony.

## Background

The purpose of assisted mechanical ventilation (MV) is to ensure adequate gas exchange and reduce work of breathing, while matching the patients' spontaneous demand in terms of timing and support [1, 2]. However, the latter cannot always be adequately achieved, leading to mismatching between subjects' timing/demand and ventilators' supply, termed as subject-ventilator asynchrony (SVA) [3]. This mismatching can occur when a mechanical ventilator automatically initiates a cycle, delays sensing of patients effort, or delivers pre-set—yet outdated and inadequate—tidal volumes, causing increased and repeated subject's inspiratory efforts [4–6]. Especially, since technical solutions are widespread, implemented in most ventilator models, and very advanced in terms of flow and pressure sensing, pre-processing and triggering, the highest incidence is seen in periods of pro-longed ventilation without close monitoring of the ventilator settings in non-adaptive ventilation modes.

Various types of SVA have been described in the literature: ineffective effort (IE), auto-triggering (AT), double-triggering (DT), reverse-triggering, delayed triggering, premature cycling, flow starvation, and excessive flow [3]. To quantify SVA incidences, the asynchrony index (AIX), computed as number of asynchrony events divided by overall number of respiratory cycles, was utilized in most studies [3]. Clinically, SVA has been encountered during invasive and non-invasive MV, with an overall incidence of up to 85% [7, 8].The SVA types with the highest incidence and clinical relevance are (I) Ineffective effort (IE), in which the subjects' inspiratory effort fails to trigger ventilator inspiration; (II) Auto-trigger (AT), in which ventilator delivered a mandatory cycle initiated without subjects' inspiratory trigger; and (III) double trigger (DT), also known as breath stacking [9] or demand mismatch, in which two consecutive inspirations stack without considerable expiration or within an short interval (less than 50% of the mean inspiratory time) [1, 6, 10, 11]. Among 62 investigations on SVA over the last 25 years, ineffective-trigger was discussed in 92% of the studies, while auto-triggering and double-triggering were found in 73% of the publications each [12].

SVA has been shown to be associated with worse clinical outcomes, such as patient discomfort [13, 14], anxiety/fear [15, 16], delirium [17], longer duration of mechanical ventilation [18], prolonged intensive care unit (ICU) and hospital stay, and higher mortality [19, 20]. Yet, links of SVA with microbiological or cellular level have not been investigated, despite almost 25 years of research.

Currently, most of the research based on clinical observations focused on the prevalence of SVA and its association with lung injury and possible diaphragm dysfunction [14, 21]. These insights led to the hypothesis that SVA might cause alveolar and organ dysfunction [22, 23]. For example, the double-triggering resulting in high tidal volume may promote ventilator-induced lung injury (VILI) in patients [24]. Furthermore, a causal relationship between SVA and worse clinical outcomes has been challenged [25, 26]. However, this cannot be elucidated in clinical research since reproducibility of SVAs and tissue sampling is limited. Additionally, there is no method to reliably and reproducibly induce SVAs in large animal experiments, in which, due to close monitoring of the appropriateness of the ventilator settings, SVAs are rare.

The aim of this investigation was to develop and evaluate a reproducible method to artificially induce SVA in an experimental lung injury model in pigs. It was hypothesized, that: (1) SVA can be artificially and reproducibly induced by alterations of ventilator parameters, and (2) different types of asynchrony can be reliably induced depending on the altered ventilator parameters.

For development and selection of suitable and reproducible modifications of respiratory parameters, bench tests on an active test lung were performed. Selected modifications were evaluated and adjusted in three pilot experiments in pigs. The modifications found most promising were then applied in a randomized two arm experimental study on pigs.

## Methods

The following types of asynchrony were sought to be induced by modification of ventilator parameters I) a mandatory cycle initiated without a prior decrease in the airway and oesophageal pressure (Auto-triggering—AT) [3, 27, 28]; II) decrease in the airway and oesophageal pressure tracing not followed by an assisted ventilator cycle (Ineffective effort—IE) [10, 18, 19, 27, 29]; and III) two consecutive respiratory flow cycles/efforts persist in close succession with incomplete expiration in between, resulting in an increased tidal volume due to the occurrence of the second breath stacking on top of the previous one (Double-triggering/breath-stacking—DT) [30, 31].

To this end, the implementation of the ventilator controller was, firstly, tested with regard to technical reproducibility in bench tests performed on an active lung simulator. Secondly, the mechanical ventilator modifications were tested for reproducibility in pilot experiments in the target animal model and the induction of DT as well as its dependence on the sedation level was tested. Thirdly, the long term reproducibility in a longitudinal experimental study was studied.

### Bench tests

**Fig. 1:**
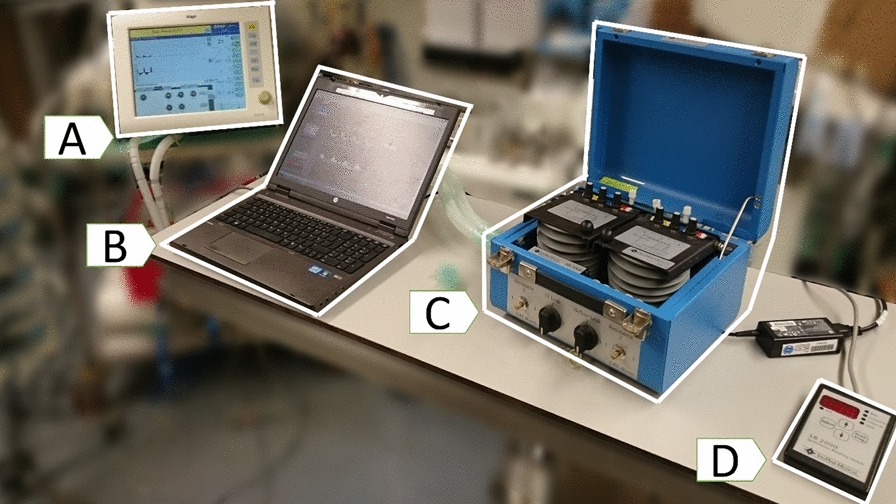
Set-up for the bench tests. Mechanical ventilator Dräger Evita XL (device **A**), laptop computer with controller software (device **B**), active lung simulator (device **C**), and spontaneous breathing module (device **D**)

Bench tests were performed on an active lung simulator (Adult/Pediatric Demonstration Lung Model, IngmarMedical Ldt., Pittsburgh, PA, USA) for IE and AT, in exemplum. Using the Evita Remote extension for the Draeger EvitaXL ventilator (Drägerwerk AG & Co. KGaA, Lübeck, Germany) the following per cycle modifications were tested to induce asynchronous cycles: ineffective effort (IE) and auto-triggering (AT). Signal acquisition from the ventilator and modification of ventilator settings was implemented in a custom-made interface developed in LabView (National Instruments, USA) [32]. The experimental set-up is depicted in Fig. 1 showing the mechanical ventilator, the active lung simulator and the Spontaneous Breathing Module SB 2000 (IngmarMedical Ldt., Pittsburgh, PA, USA).

The initial ventilator and lung simulator setting were Biphasic Positive Airway Pressure (BIPAP) mode, low pressure (P_low_) = 0 cm H_2_O, high pressure(P_high_) = 5 cm H_2_O, inspiratory to expiratory ratio I:E = 1:1, respiratory rate(RR) = 30 min^−1^, RR of the lung simulator (mimicking spontaneous breathing) = 30 min^−1^. IE and AT were performed under following settings: a) Ineffective effort: P_high_-0 cm H_2_O; b) Ineffective trigger: end-expiratory hold for one second; c) Inspiratory time = 0.7 s and RR = 45 min^−1^(temporary increase of ventilator’s RR to 1.5 times of average RR).

### Animal experiments

All experiments were performed in female pigs (German Landrace). The protocol was approved by the Institutional Animal Care and Welfare Committee and the Government of Saxony, Germany (file: DD24.1–5131/394/76) and the Animal Research: Reporting of In Vivo Experiments (ARRIVE) guidelines were followed. All experiments were divided in a set of pilot experiments to confirm the results found in the bench tests, as well as to test the induction of DT as well as its dependence on sedation levels. Following the pilot experiments, the longitudinal study was performed confirming the long term reproducibility of the induced SVAs.

### Pilot experiments

Three pilot experiments (P1, P2 and P3) were performed to elucidate means of inducing DT as well as to confirm the induction of AT and IE by alterations of ventilator parameters as found in the bench tests. Three animals (49—58 kg) were anesthetized (ketamine 15 mg kg^−1^ h^−1^, midazolam 1 mg kg^−1^ h^−1^), paralyzed (atracurium 3 mg kg^−1^ h^−1^), intubated (8.0 endotracheal tube intubation in supine position, with local anaesthesia of 2% Xylocaine spray) and ventilated with baseline settings (IPPV mode: tidal volume VT = 6 mL/kg, fraction of oxygen FiO_2_ = 1.0, PEEP = 8 cm H_2_O, I:E = 1:1, RR = 15–35 min^−1^ to achieve PaCO_2_ = 35- 45 mm Hg, inspiratory flow = 35 l min^−1^; Evita® XL, Dräger, Lübeck, Germany). Lung injury was induced by oleic acid infusion (0.5 mL/kg, aiming at PaO_2_/F_i_O_2_ < 200 mm Hg) followed by ventilator-induced lung injury (60 min of airway pressure release ventilation with pressure ramp 0.2 s, RR = 15 min^−1^, I: E = 1:1, driving pressure ΔP = 30 cm H_2_O, PEEP 0 cm H_2_O). After the muscle paralyzing agent was stopped and spontaneous breathing was resumed (25% reduction from baseline dose, except for tests of sedation levels), asynchrony was tested under following conditions, with each test and recording was implemented in a randomly selected period of 600 s:

- Test 1 – Manual holds for inducing IE with different modes in P1: BIPAP mode, PEEP = 10 cm H_2_O, P_insp_=20 cm H_2_O, I:E ratio= 1:1, RR =40 min^-1^; IPPV mode, PEEP=10 cm H_2_O, VT=350 mL (6 mL/kg), I:E ratio= 1:1, RR =40 min^-1^;
- Test 2 – Optimizing end-expiratory hold time for triggering IE in P2: BIPAP mode, PEEP = 10 cm H_2_O, P_insp_=23 cm H_2_O, I: E ratio= 1:1, RR =30 min^-1^;
- Test 3 – Dose dependency tests for IE and DT in P2 and P3: To test whether provoking IE could be done dose-dependently, three groups of tests were set with the expected pre-set incidents to be of 10%, 20%, and 30% out of the total cycles. The ventilator settings are: BIPAP mode, PEEP = 10 cm H_2_O, P_insp_=20 cm H_2_O, I:E ratio= 1:1, RR =40 min^-1^, IPPV mode, PEEP=10 cm H_2_O, VT=350 mL (6 mL/kg), I:E ratio= 1:1, RR =40 min^-1^;
- Test 4.1 – Induction of DT by setting inspiratory time to 2 s in P3: BIPAP mode: PEEP=10 cm H_2_O, P_insp_=20 cm H_2_O, I:E ratio= 1:1, RR =30 min^-1^;
- Test 4.2 – Ramp prolongation in P3: BIPAP mode, PEEP =5 cm H_2_O, P_insp_=20 cm H_2_O, RR =20 min^-1^, pressure ramp= 0.2 or 1.5 s.
- Test 5 – Effects of sedation level (P3): Light sedation group: sedation remained at 75% of the baseline sedation level; Deep sedation group: sedation remained at 85% of the baseline sedation level. The ventilator was set to BIPAP mode, PEEP=5 cm H_2_O, P_insp_=22 cm H_2_O, I: E ratio=1:1, RR = 30 min^-1^.

After six hours of experimental ventilation, animals were killed by intravenous bolus injection of 2 g thiopental and 50 mL 1 M potassium chloride.

### Experimental study

**Fig. 2:**
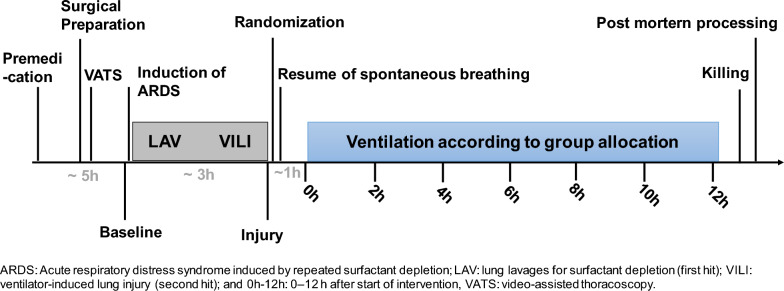
Timeline of interventions and measurements

Fourteen female landrace pigs (40–45 kg) were included in this retrospective analysis of a prospective study [33]. In brief, anaesthesia was induced and maintained using ketamine 15 mg kg^−1^ h^−1^, midazolam 1 mg kg^−1^ h^−1^, atracurium 3 mg kg^−1^ h^−1^. Fluid balance was managed using crytalloids (E-153 at 10 mL kg^−1^ h^−1^). Pigs were ventilated with a tidal volume of 6 mL kg^−1^, PEEP of 5 cm H_2_O, I:E ratio of 1:1, and F_I_O_2_ 1.0. Respiratory rate was adjusted to maintain an arterial pH of 7.35 to 7.45, and inspiratory flow was set to 35 l min^−1^ (Evita XL, Dräger, Germany). Subsequently, pigs were instrumented by surgical procedures under sterile conditions as described previously [33].The timeline of interventions is shown in Fig. 2. Following induction of lung injury via repeated surfactant depletion, two groups of pigs were included into the final analysis of this sub-study (n = 7 / group): (1) Spontaneous breathing with externally induced, high SVA (ASY group); (2) spontaneous breathing without externally induced SVA (SYN group). Sedation dose was reduced by 25% (vs. baseline), and the muscle paralyzing agent was stopped. All animals were ventilated in Pressure Assist-Control mode with PEEP = 10 cm H_2_O, P_high_ for VT = 6 mL kg^−1^, I:E = 1:1, RR = 20–35 min^−1^ for pH > 7.2, and flow-trigger of 3 L min^−1^ as well as airway occlusion pressure (airway pressure 100 ms after onset of inspiratory effort—P_0.1_) ≈ 2.5 cm H_2_O. The intervention time was 12 h from 6 p.m. In the ASY group, each modification was randomly applied in 5% of all breaths, aiming at the gross AIX (percentage of asynchronous cycles) of approximately 25%. In the synchrony group, cycle-by-cycle alterations of ventilator parameters were performed.

### Classification and quantification of asynchrony

**Fig. 3:**
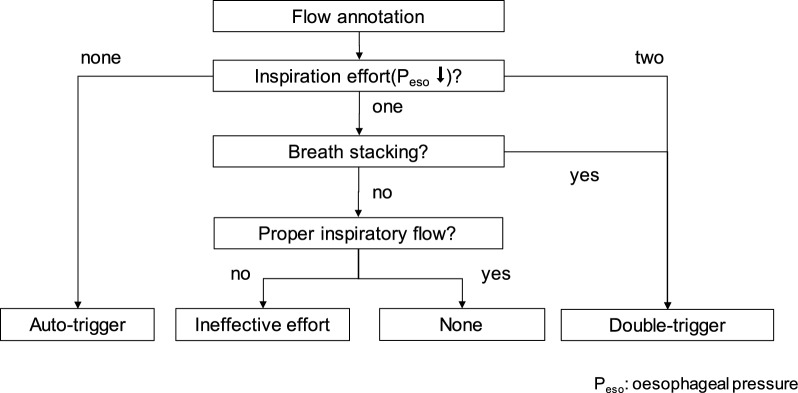
Flow chart for manual subject-ventilator asynchrony classification

Respiratory signals, including airway flow, airway pressure, oesophageal pressure, and pleural pressure, were acquired every two hours (measurements points T0 to T6) for ten consecutive minutes using custom-made LabVIEW-based software (National Instruments, USA) [34]. Respiratory cycles inspiratory and expiratory periods were annotated offline semi-automatically (MATLAB R2019a, MathWorks, Inc., Natick, MA, USA). Reference annotation of asynchronies was performed manually, by analysing the changes of airway flow, as well as airway, oesophageal, and caudal pleural pressure signals considering three adjacent respiratory cycles using VGG Image Annotator (VIA) [35]. SVA classification was performed according to predefined procedure (Fig. 3) through visual inspection by an experienced and specially trained intensive care physician. SVA classifications were exported as CSV files for statistical analysis. SVA incidence was calculated and reported as AIX as well as relative incidence for each SVA sub-type.

### Statistical analysis

Data are presented as mean ± standard deviation or median [inter-quartile range], as appropriate following Shapiro–Wilk test for normality. Data in bench and pilot tests were analysed descriptively and with graphical representation.

In experimental tests, statistical analysis of AIX and respective sub-types was carried out by two-way mixed effects repeated measures ANOVA with time (T0-T6) as with-in subject and group (ASY/SYN) as between-subject factors after application of appropriate transformation to ensure sphericity. Analyses were performed with R statistical programming language software and JASP [36]. Significance was accepted for P < 0.05.

## Results

### Bench tests

**Fig. 4:**
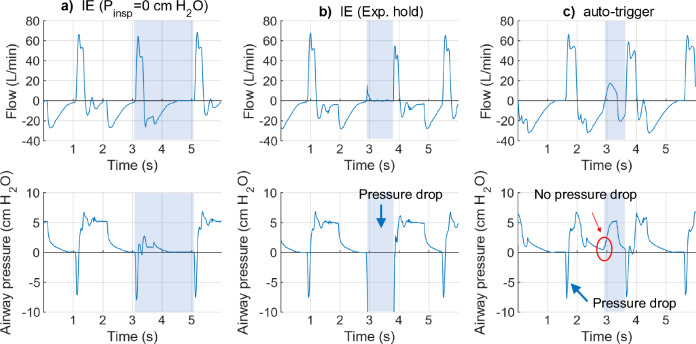
The pressure–time and flow-time waveform of provoking IE and AT at the lung simulator. Induction of IE by setting P_insp_ to zero (**a**) (the cycle from the 3rd to 5th seconds, indicated by the grey column) or by expiratory hold in (**b**) (indicated by the grey column).The cycle (from 1st to 3rd seconds) in (**a**) is the regular cycle without any adjustment. Compared to this regular cycle, an entire flow curve is observed. In (**b**), IE was provoked by an expiratory hold for one second. During the hold, the flow almost stays at the baseline (0 L/min), and a corresponding pressure drop below 0 cmH_2_O was spotted (blue arrow). In (**c**), AT was tested by temporarily increasing the RR (grey column). There is no pressure drop at the beginning of the testing cycle (the red circle), whereas the sharp valleys are detected in the adjacent regular cycles (arrows). IE: ineffective trigger, AT: auto-trigger, P_aw_: airway pressure, s: seconds, RR: respiratory rate

Temporarily adjusting inspiratory pressure to 0 cm H_2_O failed to stimulate IE (Fig. 4a). Although the inspiratory pressure was temporarily reduced to expiratory pressure (0 cm H_2_O) a complete cycle was present in the flow signal. Such a flow curve indicated that the ventilator continued delivering the gas or was effectively triggered by the lung simulator, which, in turn, did not match our expectations. Therefore, IE was induced in the lung simulator using end-expiratory holds (Fig. 4b). Thereby, flow was maintained at 0 L/min during the expiratory hold. Meanwhile, an additional pressure drop below the baseline was spotted during the hold. This is consistent with the definition of an “ineffective” trigger in which the subject inhaled (pressure dropped) while no airflow followed. Thus, end-expiratory holds were used to induce IE in the pilot experiments.

AT was implemented cycle by cycle in the lung stimulator by multiplication of RR by 1.5 (Fig. 4c). Thereby, the AT cycle (a mandatory breathing) was initiated between two adjacent regular cycles (assisted breathing). No pressure drop was captured at the beginning of the AT cycle, whereas a brief pressure drop was evident in synchronic cycles. The airway flow and pressure waveforms seen here was similar to the AT waveform observed at the bedside, in which patients passively received mandatory respiration due to premature inspiratory cycling by the ventilator. The bench tests showed that cycle-by-cycle modifications of ventilator parameters is reliable given the implemented technique and that SVAs IE and AT could be induced reliably. 

### Pilot experiments

**Fig. 5:**
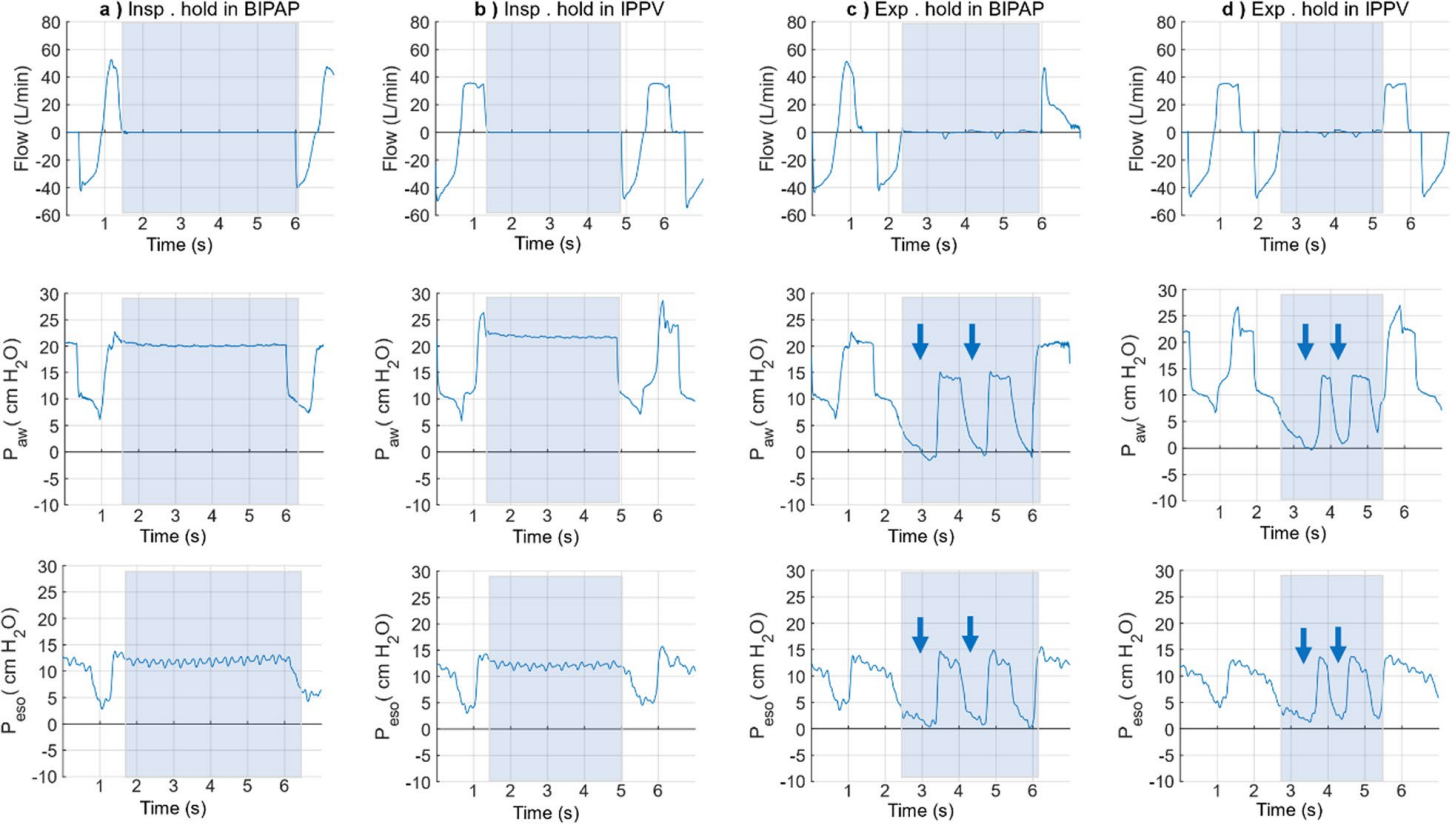
Representative flow and pressure waveforms of provoking IE by hold manoeuvres. Inspiratory hold was tested in BIPAP (**a**) and IPPV mode (**b**), and the expiratory hold was tested in BIPAP (**c**) and IPPV mode (**d**), respectively. The light blue columns indicate the respiratory holds. Initial baseline ventilation setting: BIPAP mode, PEEP = 10 cm H_2_O, P_insp_ = 20 cm H_2_O, I:E ratio = 1:1, RR = 40 min^−1^. IPPV mode: PEEP = 10 cm H_2_O, VT = 350 mL (6 mL/kg), I:E ratio = 1:1, RR = 40 min^−1^. Upward concavities below 10 cm H_2_O in pressure curves were considered active inspiratory efforts and were captured at expiratory hold phrases in BIPAP and IPPV modes (c and d, marked with arrows). The jagged waveform in P_eso_ was due to the interference with cardiac cycles and aortic pulsation. IE: ineffective trigger; BIPAP: Biphasic positive airway pressure): pressure assist/control mode. IPPV: Intermittent Positive Pressure Ventilation; PEEP: positive end expiratory pressure; P_insp_: inspiration pressure; I:E ratio: inspiratory to expiratory time ratio; RR: respiratory rate; P_aw_: airway pressure; P_eso_: oesophageal pressure; and VT: tidal volume

IE was reproducibly induced in pigs by using end-expiratory, but not by inspiratory holds (Fig. 5). During inspiratory hold, airway pressure remained constant at the peak level (20 cm H_2_O) without any inhale-related drop during the inspiratory hold (Fig. 5a, and b). Such tracings indicated that the inspiratory hold failed to induce IE in Bi-phasic Positive Airway Pressure (BIPAP) and Intermittent Positive Pressure Ventilation (IPPV) ventilation modes. In contrast, airway pressure dropped significantly below pre-set expiratory level (10 cm H_2_O) during the expiratory hold, which implied the presence of active inspiratory efforts against a closed valve (Fig. 5c, and d). Airway flow was almost maintained at 0 mL/s, implying that the ventilator did not trigger a corresponding gas delivery.

**Fig. 6:**
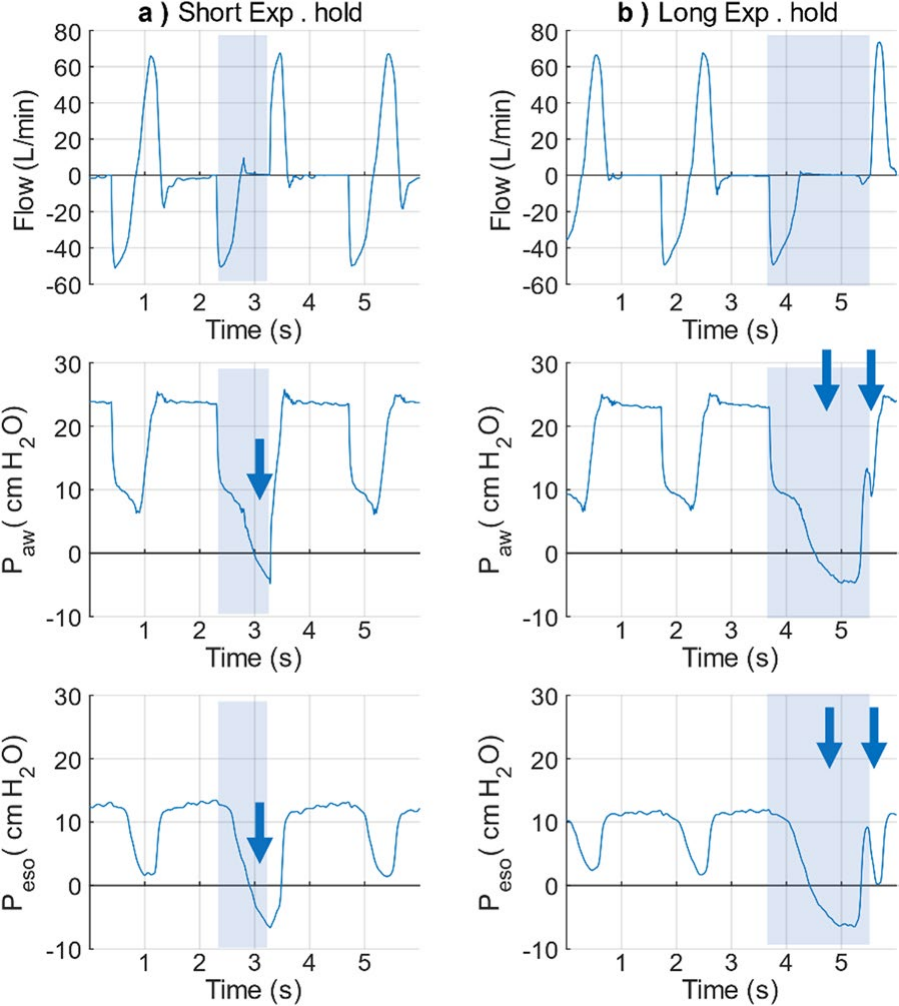
Representative flow and pressure waveforms of provoking IE by adjusting hold duration. **a**, hold for less than 1.5 fold of the regular expiratory time; (**b**), hold for more than 1.5 fold of the regular expiratory time. An incline slope of pressure below 10 cm H_2_O was captured in (**a**), followed by flow and pressure rising to the peak level directly. It suggests the ventilator started an air delivery immediately after the hold ended while the pig was inhaling. In (**b**), the airway pressure returned to nearly 10 cm H_2_O (terminate the inhaling effort) after dropping below 0 cm H_2_O before the hold ended (marked with arrows). Such typical pressure upward concavity without any flow changes implied that the pig failed to trigger the ventilation till the inhale effort ended. IE: ineffective trigger. P_aw_: airway pressure, P_eso_: oesophageal pressure

Long expiratory hold manoeuvers (> 1.5-fold inspiratory time) induced IE-like waveforms comparted to short expiratory holds (Fig. 6). With a hold of less than 1.5-fold of the expiratory time, the ventilator delivered a flow right after the hold ended during active inhalation (Fig. 6a). Under this condition, the pig managed to trigger the ventilation through a strong inspiration (the incline slope of P_aw_ and P_eso_ below 10 cm H_2_O followed by flow rising to the peak level in Fig. 6a), which is the opposite of the IE definition. Instead, an upward concavity in the P_aw_ and P_eso_ curve was obtained, typical for IE, when the hold was prolonged to more than 1.5-fold of the expiration time (Fig. 6b).

An IE > 5% was possible to induce in BIPAP and in IPPV ventilation modes (9.1 ± 5.11% in BIPAP mode, 5.6 ± 0.57% in IPPV mode), respectively. Moreover, dose dependent IE could be induced during BIPAP (Table 1). In IPPV, 12.0%, 23.5%, and 30% IE were obtained for pre-set IE incidence of 10%, 20%, and 30%, respectively.

**Table 1:** AIX of IE and DT in dose-dependent tests in BIPAP modes

Set AIX of IE/DT	AIX of IE (%)	AIX of DT (%)
10%	13.2%	8.0%
20%	21.6%	18%
30%	30.3%	15%

*AIX* asynchrony index, *IE* ineffective triggering, *DT* double-triggering, BIPAP (Biphasic positive airway pressure): pressure-assist/control mode

**Fig. 7:**
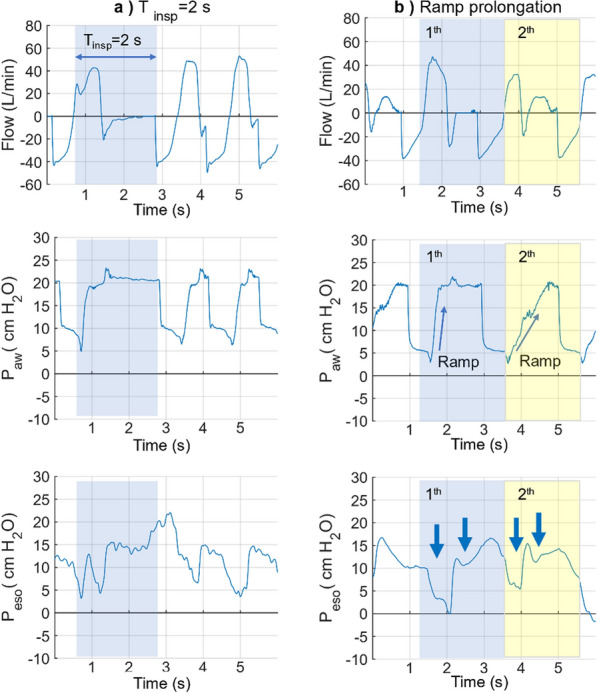
Representative flow and pressure waveforms during provocation of DT. **a** by readjusting T_insp_ to 2 s and (**b**) by ramp prolongation. In (**a**) (light blue column), two upward concavities pressure drops were captured in the P_eso_ curve, which indicated the active inspiration. However, part of the flow almost remained constant without the expected corresponding two peaks. In (**b**) the 1st cycle (light blue column, ramp 0.2 s), pressure rapidly increased and kept at the peak level, and there was only one peak flow in the inspiration phase. While In (**b**) 2nd cycle (light yellow column, ramp 1.5 s), pressure rose slowly, and two flow peaks were observed in the inspiration phase. The two consecutive upwards concavity P_eso_ drops were captured in both figures (arrows), which implied the active inspiration of the pig. DT: double trigger. T_insp_: inspiration time. P_aw_: airway pressure, P_eso_: oesophageal pressure

Induction of DT was implemented by adjusting the inspiration time and the airway pressure ramp prolongation (Fig. 7). One additional distinct pressure drop was captured in both P_aw_ and P_eso_ within the inspiration phase after adjusting the inspiration time to 2 s (baseline setting 1.5 s), indicating the pig’s active inspirations (Fig. 7a). These two adjacent inhalations in the inspiration phase without considerable exhalation were similar to the DT observed at the bedside.

DT was induced in both BIPAP and IPPV modes (3.1 ± 2.54% in BIPAP mode, 3.95 ± 1.06% in IPPV mode). Assuming that a prolonged airway pressure rise-time might provoke DT, BIPAP was chosen as mode of ventilation for longitudinal study. When setting the airway pressure rise-time to 1.5 s, two flow peaks were observed in the inspiratory phase (Fig. 7b 2nd cycle, “M” shape in the flow graph). These waveforms are closer to the clinical DT, than the single flow peak with a 0.2 s ramp in 1st cycle. Moreover, DT induction could be induced dose dependently (Table 1), It resulted in 8% for the pre-set 10% group, 18% for the pre-set 20% group, and 15% for the pre-set 30% group in BIPAP mode. Compared to pressure rise-time modification for one respiratory cycle, a cluster of three consecutive DT cycles yielded a higher AIX. It resulted in 18% for the pre-set 10% group, and 20% for the pre-set 20% group.

**Fig. 8:**
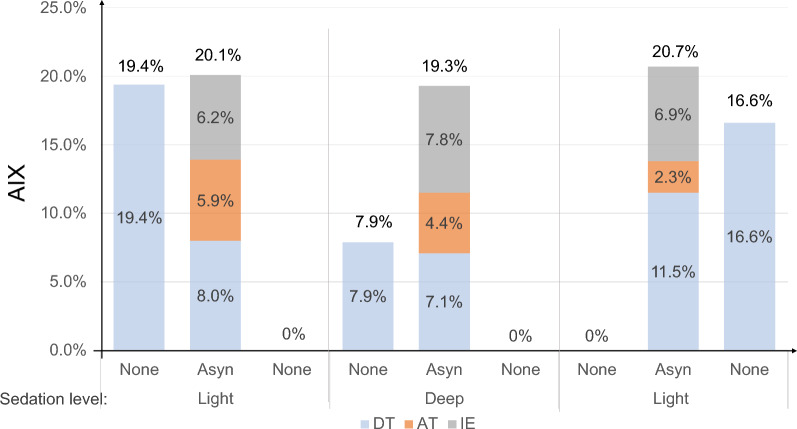
Total AIX and subtype AIX in different sedation levels. Light: sedation at 75% of baseline sedation level; Deep: sedation at 85% baseline sedation level. None: without asynchrony treatment; Async: with three subtype asynchrony provoked. AIX: asynchrony index; DT: double-triggering, IE: ineffective effort, AT: auto-triggering

Total AIX during light sedation and asynchronous ventilation (20.1% and 20.7%) was similar, yet slightly higher, than during deep sedation (19.3%). This seemed to be unspecific to pre-set AIX because DT was 19.4% and 16.6% in two intervals during conventional synchronic ventilation, while being zero in the other two intervals. AIX of EI and AT were higher than 5% during light sedation and asynchronous ventilation and were zero during conventional ventilation (Fig. 8). As a consequence of the generally higher AIX yet less specific induction of DT, the light sedation group (75% of the baseline sedation) was chosen for the experimental study.

The pilot experiments confirmed the reliable induction of AT and IE, by temporary increase of RR and end-expiratory holds, respectively. The induction of DT was confirmed to be implemented in the most reliable way by increasing the pressure rise-time during BIPAP mode in three consecutive cycles, however, was shown to be independent on sedation level. The induction of all three types of SVA was independent of the tested sedation levels.

## Experimental study

**Fig. 9:**
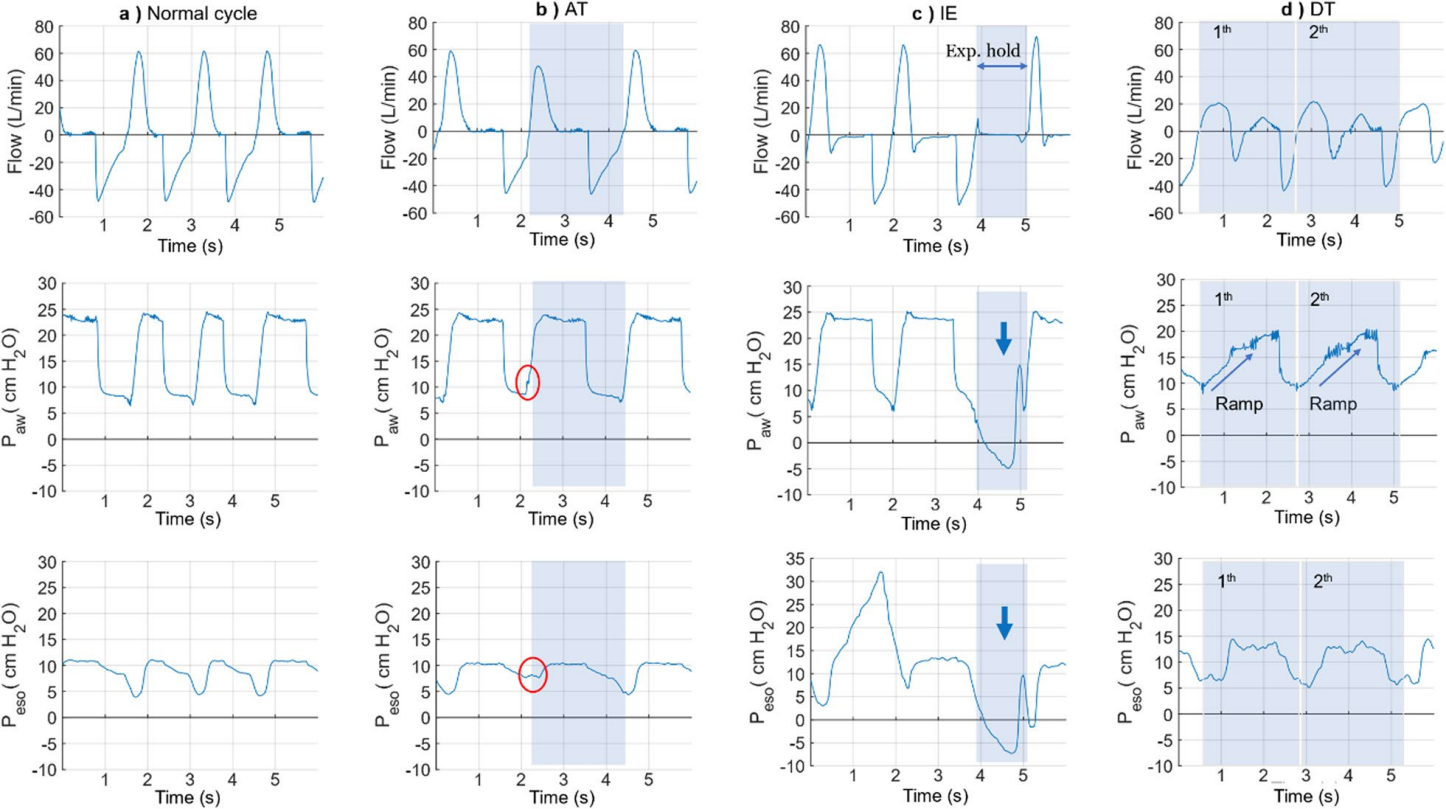
Representative tracings of a normal breathing cycle and each SVA sub-type (light blue column). With the flow, airway pressure, and oesophageal pressure recordings, respectively. **a** normal breathing cycle with the inspiration and expiration phases. **b** auto-trigger (AT), P_aw_ increase without a corresponding dip (spontaneous inhaling) right before the breathing cycle. Instead, a tiny dip (near the start of inspiration) was detected before the Paw rising (red circle), which indicated that the ventilator started a mandatory delivery directly. **c** Ineffective Effort (IE), an upward concavity below the pre-set 10 cm H_2_O (arrow) was detected in both P_aw_ and P_eso_ while flow remained 0 mL/s during the manual expiratory hold. Such pressure drops indicated the subject tried to inspire and was forced to terminate the effort due to no airflow delivered. **d** Double Trigger (DT), two pressure drops (upward concavity) were captured during the prolonged inspiration phase in both P_aw_ and P_eso_, which implied two inspiration efforts. Meanwhile, two consecutive positive flow curves (value above 0 mL/s) were captured during the inspiration (“M” shape in the flow graph), implying the ventilator delivered twice accordingly. P_aw_: airway pressure; P_eso_: oesophageal pressure

Example tracings of AT, IE, DT, and normal-cycle tracings in our experimental study are shown in Fig. 9. Instead of a large pressure drop before airway pressure increases, a tiny pressure dip was captured in the representative AT cycle, indicating that the ventilator started a mandatory delivery without an inspiratory trigger (Fig. 9b). A representative IE tracing was consisting of inspiratory effort in both P_aw_ and P_eso_ (an upward concavity below the pre-set 10 cm H_2_O) without an increase in inspiratory flow or airway pressure (Fig. 9c). Representative DT waveforms with a prolonged inspiratory phase are shown in Fig. 9d.

Based on the results from the bench and pilot tests, SVA was induced in the subsequent experimental study by applying cycle-by-cycle variations of mechanical ventilation in the ASY group as follows: For AT cycles, inspiratory time was set to 0.5 s and increased RR by 1.5 times. For IE, the duration of automatic expiratory hold manoeuvres was adjusted to 1.5-fold inspiratory time. DT was induced by prolonging the pressure ramp (temporarily altering the pressure ramp interval to 2 s) for three consecutive cycles.

**Fig. 10:**
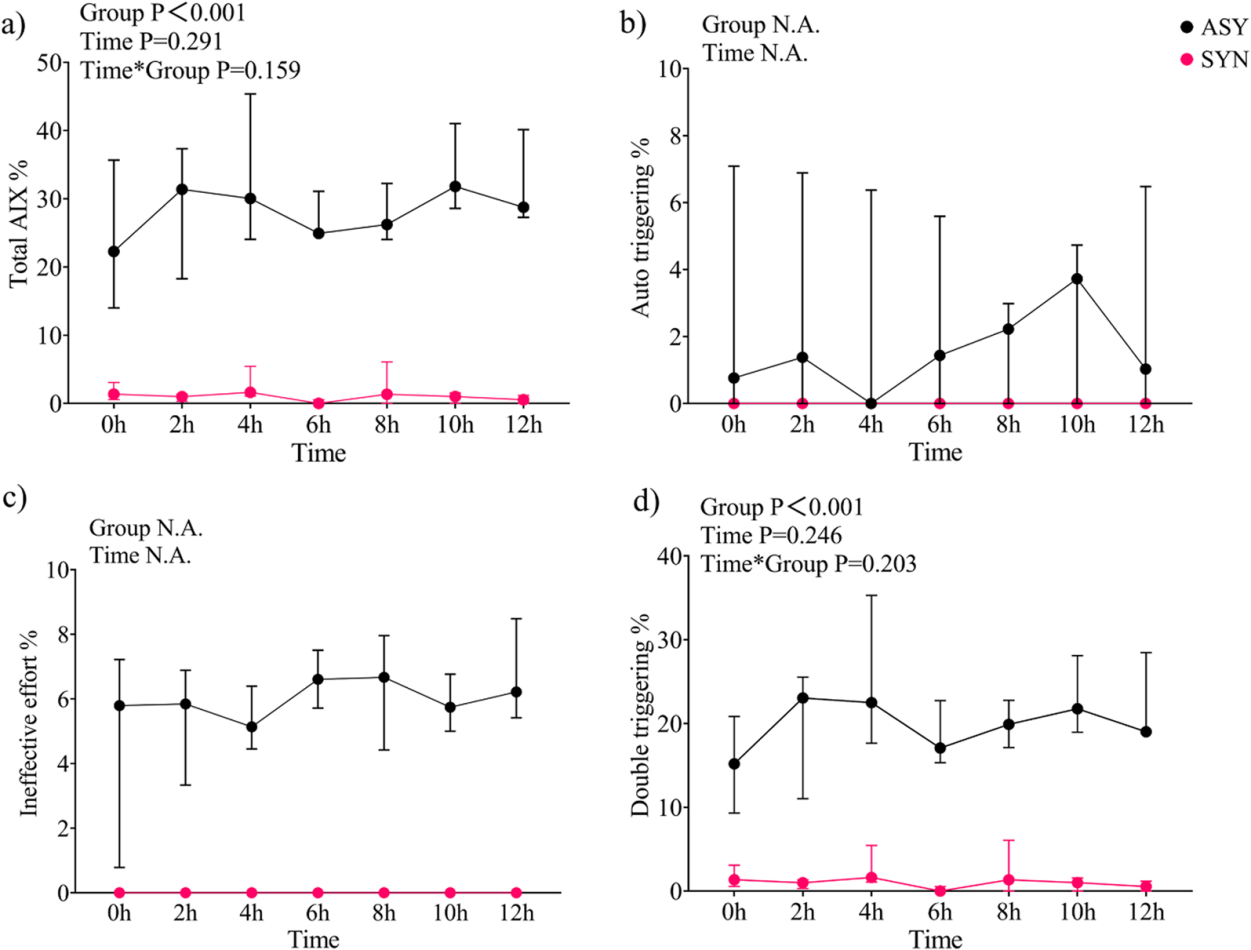
Total asynchrony index (**a**), percentage of auto-triggering (**b**), ineffective effort (**c**) and double trigger (**d**). Values in median [IQR] percentage of total number of cycles; 0 h-12 h after start of intervention; AIX: Asynchrony Index. *ASY* Asynchrony group, *SYN* Synchrony group, for a detailed description refer to Statistical analysis subsection in Methods

Overall, AIX was dependent on the intervention group (P < 0.001, Fig. 10a) but did not show to be dependent on time (P = 0.291) nor on intervention-time mixed effects (P = 0.159). The median of total AIX was 28.8% [24.0%–34.4% interquartile range] and 1.0% [0%–1.6%] in the ASY and SYN groups (0-12 h), respectively (Fig. 10a). Incidence of AT cycles was 2.4% [0%– 5.8%] in the ASY group, whereas 0% AT was found in the SYN group (Fig. 10b). Incidence of IE was 5.9% [5.0%- 6.9%] in the ASY group, while the AIX was 0% in the SYN group (Fig. 10c). DT incidence was higher in the ASYN 20.0% [16.2%– 24.2%] compared to SYN group 1 in which it was 0% [0%– 1.6%] (P < 0.001), with neither time nor intervention-time mixed effects, while there was no time effect (P = 0.246) and no group-by-time interaction (P = 0.203) (Fig. 10d).

## Discussion

The main results of this research maybe summarized as: 1—SVA may reproducibly be induced by cycle-by-cycle modification of ventilator parameters; 2—Reproducibility seemed to be dose-independent but depended on the type of asynchrony; 3—Combined induction of three investigated SVA types allowed to induce AIX > 15% in an experimental translational study in a model of acute lung injury in pigs [33].

In clinical scenarios, IE and AT may occur when the respective trigger is not titrated to the patients respiratory drive [3, 37–40]. A trigger threshold level set too high may result in failure to trigger the mechanical ventilator and, thus, in an ineffective effort. A trigger threshold level set too low may results in automatic triggering of the ventilator, e.g. by artefacts in the respective flow signals caused by air leaks, movement of the ventilator tubing, humidifiers, valves or even of the patient. Although it would have been possible to alter the respective trigger threshold on a cycle-by-cycle basis, appropriate levels to trigger IEs or ATs would have been subject to time-variant respiratory muscle function and respiratory drive of the animals.

The induction of AT by initiating a controlled cycle slightly earlier than the next expected respiratory cycle, allowed to reproducibly generate AT, that closely resemble the clinical situation of the patient [39, 41, 42], being subject to purely controlled and possibly unrequested inspiration that will lead to an inspiratory effort on a high airway pressure level and thus extensive stress to the parenchyma and respiratory muscles. It has to be noted that, in clinical settings, the incidence of AT was associated with long expiratory time intervals [41]. However, the authors regarded this association being caused by longer zero flow times, increasing the chances that random artefacts in airway flow trigger the mechanical ventilator.

The way that IE was induced here, by end-expiratory holds not only allowed for a controlled and reproducible provocation of IE but also mimicked the extensive respiratory effort and resulting stress on respiratory muscles in the clinical scenarios.

In the bench test of this investigation, it was observed that setting the inspiratory pressure equal to the end-expiratory pressure cannot stimulate IE. Despite active inspiration in P_aw_, flow still occurred due to pressure compensation in ventilator provide set airway pressure [39, 43].

In contrast typical IE waveforms were obtained by expiratory hold manoeuvers. This hold technique was then applied in the pilot pig study for IE tests in both inspiratory and expiratory phases. Surprisingly, no inhalation was observed during inspiratory hold, whereas typical inhalations with missed flow cycles occurred during expiratory hold. These results may be explained by the fact the central-nervous respiratory drive is determined by partial pressures of blood gasses or the carotid arterial chemoreceptors [44]. This involuntary mechanism senses CO_2_ changes in blood and stimulates the respiratory muscles to facilitate breathing.

Conversely, expiration is a passive action in mammals at rest. It is immediately followed by an active inspiration [45]. In our study, the active inhalation was captured in both airway and oesophageal pressure, while the flow stayed at baseline. Particularly by readjusting the hold duration to at least 1.5 times the regular expiration phase, nearly identical IE curves were observed compared to those seen at the bedside [10, 28, 29].

Furthermore, hold manoeuvers allowed for a predetermined IE incidence in the range of 0–30%. Conducting two to nine holds for IE cycles out of 30 cycles per minute is straight forward. Also, it is possible to perform the IE cycles intermittently or consecutively.

Although DT definitions and typical wave forms vary in publications, most researchers agree on its defining feature: two inhalations occurring within one inspiration phase of the ventilator with or without a short expiration interval [30, 37, 46, 47]. This phenomenon is characterized by an "M" profile in the flow curve, while a regular flow waveform only has one peak [28, 38]. In our experiments, the DT flow curve induced by ramp prolongation could only simulate short exhalations (a tiny valley) between two consecutive inhalations instead of the relatively long exhalations in other projects [42, 48].

We assumed that prolongation of inspiration time might disturb the synchronization between the ventilator and the pig. DT was tested with an increased inspiration phase of 1.5 s. As a result, two active inhalations were observed during inspiration, reaching above 5% in BIPAP mode. However, there was no typical "M" shape in the flow curve. We then considered the modification of the ramp to provoke double flow peaks. Setting a long ramp allows the ventilator to start at a lower pressure and gradually increase it over a pre-set period. It is expected that a prolonged 1.5 s ramp would be longer than the pig's inspiration and therefore induce DT. As a result, the 1.5 s ramp caused the pig to breathe twice during the slow increase of the pressurization. In contrast, the 0.2 s ramp forced the pressure to reach the platform pressure quickly, resulting in a single flow peak like other regular cycles. This “M” shape flow curve with a short expiration interval is consistent with the flow waveform during DT in other publications [28, 38, 42]. However, in a classical DT scenario, only the first cycle is subject-triggered due to the conflict between the subject’s high demand and the relatively short ventilator inspiration time [30, 37]. This definition means one subject’s effort along with two inspiration flow cycles.

In this study the authors considered double triggering a.k.a. immature cycles are caused by a demand mismatch between the patients effort and the delivered ventilator support causing reduced (speed of) alveolar filling. In theory, this could be provoked by modification of the inspiratory time, a too low totally generated tidal volume, or an insufficient early response by the ventilator. Given the model design requirement, that cycle-by-cycle tidal volume and plateau airway pressure sought to be constant, independent of the modified ventilator parameters, we opted to simulate an insufficient early response by the ventilator by increasing the pressure ramp time.

In the longitudinal study, overall AIX was greater than 15% in the asynchrony group, aligning with the asynchrony definition in publications [3, 27]. In the synchrony group, in accordance with the authors experience with animal experiments, incidence of AIX was zero for AT and IE and below 2% for DT. This is mainly explained by the well-developed ventilation protocol and close monitoring of the ventilator settings during the animal experiments. Auto-triggering in the asynchrony group was less than the set 5% in two pigs, possibly due to high spontaneous respiratory rate, which is common in pigs with acute lung injury. Nonetheless, our results reproduced the clinical asynchronous phenomenon that DT and IE occurred most frequently [3, 28]. In addition, our results showed no time effects for each type of asynchrony as well as overall AIX although studies in humans and animals reported difference central breath variation in daytime and night-time [49–52]. However, the pigs in our study were sedated from six pm to six am the next day, which may have only overlapped partly with diurnal rhythm. Although not significant a slight drop in incidence of DT might have been present, however not relevant compared to AIX variance induced by other factors between animals.

Our study has implications for research on pathophysiology caused by asynchrony which were not possible to study due to a lack of appropriate animal models. By investigating the effects of different types and levels of asynchrony on organ function and injury, our model may improve the management of critically ill patients on mechanical ventilation, particularly in patients with acute respiratory distress syndrome (ARDS). The knowledge gained with this experimental tool may thus help mitigate associated pulmonary complications as well as ventilator-induced lung injury. Moreover, our study provides a platform to train ICU clinicians in recognizing asynchrony and managing mechanical ventilation in a highly simulated environment. In addition, this platform can contribute to testing efficacy and safety for novel ventilators and bedside devices. For example, the method described here may be used to develop algorithms to detect and classify types of asynchrony based on non-invasive measurements techniques as e.g. opto-plethysmography, enabling the distribution of muscle activity [53–55], or electrical impedance tomography to measure tidal volume distribution dynamics, including Pendelluft [56], especially in the advent of 3D-EIT [57].

To date our study is the first to establish a pig model for induction of three major SVA types using modification of ventilator setting on a cycle-by-cycle basis, describing the development from bench tests to experimental animal models. In one recent publication SVA was induced by phrenic nerve stimulation in a model of ARDS in rabbits [58]. Therein, the authors focused on investigating the potential lung and diaphragm injury caused by SVA by simulating breath-stacking and reverse-triggering for a period of four hours of ventilation, with assist controlled ventilation without inducing SVA as the control group. Using phrenic nerve stimulation, the authors achieved incidences of breath stacking and reverse triggering as high as 92 ± 12% and 50 ± 5%, respectively [58]. Ineffective efforts and auto-triggering were not considered. Phrenic nerve stimulation also represents an interesting and seemingly reliable approach. However, this method requires bilateral surgical identification and preparation of the phrenic nerves as well as their direct electrical stimulation using nerve block needles and a stimulator under continuous cooling to prevent nerve injury. As such, this method is more labour-intensive and invasive and, thus, carries significantly higher risks than the method proposed by our group. Furthermore, the animals´ spontaneous breathing was completely suppressed by deep sedation. In contrast, our model allowed lighter sedation and preserved spontaneous breathing, likely allowing broad applicability and increasing clinical relevance.

Pigs were chosen to develop the asynchrony model for the following major reasons. First, the anatomical structure, physiology, and immune system of pigs resemble that of humans [59, 60]. Second, the pig lung is very similar to a human lung, represented by the structure of alveoli and bronchioles, chest wall, and relevant lung mechanics. Third, pigs can tolerate extended periods of mechanical ventilation compared to laboratory rodents (e.g., mice), allowing to study effects of prolonged mechanical ventilation [61]. Therefore, a pig model is suitable for evaluating mechanical ventilation and respiratory support devices to ensure experimental safety and performance [62–66]. Its weight and tissue size allows for the application of most of the techniques designed for humans, such as the oesophageal balloon catheter.

This study knows several limitations. Firstly, this study was conducted in two different models of lung injury, namely surfactant depletion and oleic acid injection, which may not completely reflect the clinical representation of lung injury. Secondly, the main experiment was conducted over twelve hours. While this time frame is closer to clinical practice than many prior animal studies, it may still be insufficient to capture the full range of physiological effects of SVA, although the high respiratory rate in pigs results in more events overall than a comparable period in clinical patients.

## Conclusion

Three types of subject-ventilator asynchrony with the highest incidence, namely ineffective effort, auto-triggering, and double trigger, can be reproducibly induced by expiratory holds and modification of respiratory rate and inspiratory pressure slope. Applying this method to a surfactant depletion-based model of lung injury in pigs may be a feasible, reliable, and reproducible model for future research on asynchrony.

## Data Availability

No datasets were generated or analysed during the current study.

## Abbreviations

AIX: Asynchrony index
ARDS: Acute respiratory distress syndrome
AT: Auto-triggering
BIPAP: Biphasic positive airway pressure
DT: Double-triggering
ICU: Intensive care unit
IE: Ineffective efforts
IPPV: Intermittent positive pressure ventilation
LAV: Lung lavage for surfactant depletion
MV: Mechanical ventilation
RR: Respiratory rate
SVA: Subject-ventilator asynchrony
VIA: VGG image annotator
VILI: Ventilator-induced lung injury
